# Antifeedant Effects and Repellent Activity of Loline Alkaloids from Endophyte-Infected Tall Fescue against Horn Flies, *Haematobia irritans* (Diptera: Muscidae)

**DOI:** 10.3390/molecules26040817

**Published:** 2021-02-04

**Authors:** Javier Espinoza, Manuel Chacón-Fuentes, Andrés Quiroz, Leonardo Bardehle, Paul Escobar-Bahamondes, Emilio Ungerfeld

**Affiliations:** 1 Laboratorio de Ecología Química, Departamento de Ciencias Químicas y Recursos Naturales, Universidad de La Frontera, Casilla 54-D, Avenida Francisco Salazar 01145, Temuco 4811230, Chile; manuel.chacon@ufrontera.cl (M.C.-F.); leonardo.bardehle@ufrontera.cl (L.B.); 2 Centro de Excelencia en Investigación Biotecnológica Aplicada al Medio Ambiente (CIBAMA), Universidad de La Frontera, Avenida Francisco Salazar 01145, Temuco 4811230, Chile; 3 Departamento de Producción Agropecuarias, Facultad de Ciencias Agropecuarias y Forestales, Universidad de La Frontera, Casilla 54-D, Avenida Francisco Salazar 01145, Temuco 4811230, Chile; 4 Instituto de Investigaciones Agropecuarias (INIA), Centro Regional de Investigación Carillanca, Vilcún, Región de La Araucanía, Temuco 7500502, Chile; paul.escobarc@inia.cl (P.E.-B.); emilio.ungerfeld@inia.cl (E.U.)

**Keywords:** loline alkaloids, lolines, horn flies, *Haematobia irritans*, antifeedant agents, repellent effect

## Abstract

*Haematobia irritans* is an obligate bloodsucking ectoparasite of cattle and is the global major pest of livestock production. Currently, *H. irritans* management is largely dependent upon broad-spectrum pesticides, which lately has led to the development of insecticide resistance. Thus, alternative control methods are necessary. Endophyte-infected grasses have been studied as an alternative due to their capability to biosynthesize alkaloids associated with anti-insect activities. Thus, the main aim of this study was to evaluate the antifeedant and repellent activity of lolines obtained from endophyte-infected tall fescue against *H. irritans* adults in laboratory conditions. The alkaloid extract (ALKE) was obtained by acid–base extraction. *N*-formyl loline (NFL) and *N*-acetyl loline (NAL) were isolated by preparative thin layer chromatography (pTLC) and column chromatography (CC), and the loline was prepared by acid hydrolysis of a NFL/NAL mixture. Loline identification was performed by gas chromatography coupled to mass spectrometry (GC/MS). Feeding behavior was evaluated by a non-choice test, and olfactory response was evaluated using a Y-tube olfactometer. Accordingly, all samples showed antifeedant activities. NFL was the most antifeedant compound at 0.5 µg/µL and 1.0 µg/µL, and it was statistically equal to NAL but different to loline; however, NAL was not statistically different to loline. NFL and NAL at 0.25 µg/µL were more active than loline. All samples except loline exhibited spatial repellency in the olfactometer. Thus, the little or non-adverse effects for cattle and beneficial activities of those lolines make them suitable candidates for horn fly management.

## 1. Introduction

*Haematobia irritans irritans* (L.) (Diptera: Muscidae), commonly named the horn fly, is an obligate bloodsucking ectoparasite of cattle [1] and it is considered one of the major global pests of livestock production [2]. It is a little dark gray fly of between 2 and 5 mm in length, which makes it the smallest biting fly that attacks beef cattle [3,4]. It can also parasitize horses, water buffalo, sheep, goats, and some non-domesticated mammals [5,6]. *H. irritans* is widely distributed, preferentially in tropical, subtropical, and some temperate regions of the Northern Hemisphere [7], including Europe, Asia Minor, and North Africa. In the Americas, *H. irritans* ranges from southern Canada to temperate areas of Argentina, Uruguay [8,9,10,11], and Chile [11]. In 1968, some colonies of horn flies were found in Chile for the first time, but they disappeared [12]. The species was definitively established in 1993 [11,12]. Currently in Chile, *H. irritans* is found between the regions of Arica y Parinacota and Aysén and it emerges from November until May, showing a large population from December until March [12]. Both *H. irritans* sexes use their piercing proboscis to feed on cattle 24–38 times per day [5], causing annoyance and alteration of grazing behavior, especially when the animals suffer a massive infestation (>200 horn flies per animal) [13,14,15]. This provokes a reduction in feed conversion efficiency and reduced milk production and weight gain [5,15,16,17,18]. One beef cattle can be parasitized by up to 4000 horn flies and can lose approximately 0.25 kg per day [7], whilst dairy cow milk yield can decline by 20% [19,20]. In the United States, the damages caused by the horn fly cost USD 1 billion each year, including costs of chemical protection [1,5,21,22], whereas annual losses in Brazil were estimated to be between USD 150 million and USD 2.56 billion in 2012 [23]. In Chile, the annual economic losses caused by *H. irritans* were estimated at CLP 25,800 million [24,25], equivalent to USD 45 million (January 2001). This did not consider the losses produced by the decrease in reproductive efficiency and losses in leather quality [26].

Management of *H. irritans* is largely dependent on broad-spectrum pesticide use, mainly organophosphates and pyrethroids. Unfortunately, pesticides are highly persistent, harmful to the environment, they have significant residual toxicity, and have led to the development of insecticide resistance [27,28,29]. Due to this resistance and negative environmental effects, alternative control methods are necessary to deal with this pest. Over the last decade, the use of grasses infected with endophytic fungi has been proposed as an alternative [30,31]. These fungi grow in plant tissues and are capable of having a mutualistic relationship with the host. The fungi receive nutrients and protection from the host, and the host receives protective agents against hydric stress, extreme temperatures, and herbivory [32,33,34]. Fungal endophytes, mainly species from the *Neotyphodium* genus, in their symbiotic associations with grasses are able to biosynthesize up to four classes of alkaloids: ergot alkaloids, indole–diterpene alkaloids, lolines, and peramine [35], although no individual fungal isolate is known to produce representatives of all four classes [36].

**Figure 1:**
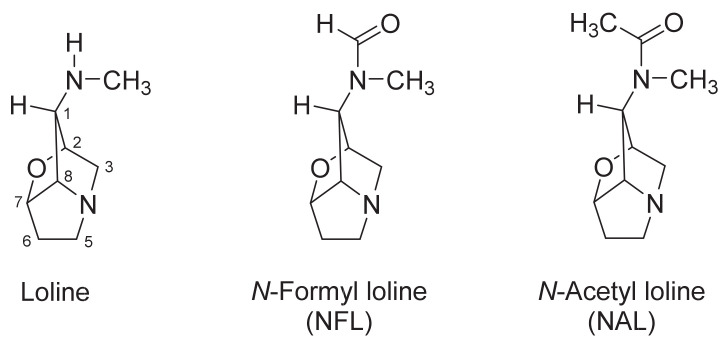
Chemical structures of loline alkaloids.

Differences in the horn fly load on cattle affected by endophyte infection of pastures have been described [30]. When cattle were grazed in endophyte-infected tall fescue pastures, a decrease in the fly load of up to 50.5% was observed [37]. Additionally, cattle dung from steers that fed on infected tall fescue was associated with an 80–90% *H. irritans* larval mortality [30,31] and only 29% adult emergence from pupae [31]. Also, lolitrem B, an indole–diterpene alkaloid, and peramine were present in the dung of steers fed on *N. coenophialum*-infected tall fescue pastures [31]. In another study, alkaloids extracted from endophyte-infected tall fescue seed containing *N*-formyl loline (NFL), *N*-acetyl loline (NAL), and loline (Figure 1) (59:21:20 by mass, respectively) caused 100% mortality of first-instar horn fly larvae when bovine dung was supplemented with the extract. When dung was supplemented with acyl loline derivatives, NFL was more toxic than NAL, whilst loline hydrochloride was not toxic [38]. In a factorial combination study, first-instar *H. irritans* larvae were exposed to heifer dung supplemented with a mixture of NFL and ergotamine tartrate (EAT). In the absence of EAT, NFL caused a dose-dependent linear decline in the number of pupae that recovered. In the absence of NFL, EAT showed significant quadratic responses in *H. irritans* larvae [39]. Despite benefits to the control of *H. irritans*, ergot alkaloids and indole–diterpene alkaloids have been related to the fescue toxicosis syndrome in livestock [40]. On the other hand, peramine and lolines have not been associated with toxic effects on cattle and they have showed insecticidal activity against a wide diversity of insects [41,42,43,44]. Despite the above, there is no information about the effect of loline alkaloids on the adult stage of *H. irritans*. Therefore, the main aim of this study was to evaluate the antifeedant effects and repellent activity of loline alkaloids obtained from endophyte-infected tall fescue against *H. irritans* adults in laboratory conditions.

## 2. Results

Fifteen steers were fed on endophyte-free tall fescue pasture (E−). Afterward, endophyte-infected tall fescue (E+), steer blood, and horn flies from steers fed on tall fescue (E−) were sampled. An orange-brown oil (0.2378 g, 0.079% *w/w* on a dry matter basis) corresponding to the alkaloid extracts (ALKE) was obtained by acid–base extraction from tall fescue (E+) that was 97% infected with *Neotyphodium coenophiaum*. A yellow oil (0.092 g) corresponding to a 1:1 *N*-formyl loline/*N*-acetyl loline mixture (NFL/NAL) was obtained from ALKE by preparative thin layer chromatography (pTLC). Two pale-yellow oils corresponding to NFL (4.8 mg) and NAL (6.9 mg) were obtained from the NFL/NAL mixture by column chromatography, and another pale-yellow oil, corresponding to loline (8.6 mg; 55.8 µmol; 35.3%), was yielded by the acid hydrolysis of the NFL/NAL mixture.

Loline alkaloid analysis and identification were performed by comparing retention times and mass spectrums from gas chromatography coupled to mass spectrometry (GC/MS) analysis with those of commercial standards. Accordingly, NFL and NAL were present in ALKE in the same proportion, corresponding to 90.14% of the total detected chromatographic signals. The remainder that was detected corresponded to a few alkaline compounds and long-chain saturated alcohols. Only NFL and NAL were detected in the alkaloid mixture in a 1:1 proportion, indicating the success of the purification process. The GC analysis of the two oils obtained from NFL/NAL mixture separation by column chromatography (CC) showed that one oil contained 95.2% NFL and the other contained 95.0% NAL, proving that NFL and NFL separation from the loline mixture was a success. In the same way, the oil obtained from the NFL/NAL hydrolysis presented 96.1% loline, indicating the success of the chemical modification. Thus, five alkaloidal samples were obtained: (a) ALKE obtained directly from tall fescue (E+), (b) NFL/NAL mixture, (c) NFL, (d) NAL, and (e) loline obtained from hydrolysis of the NFL/NAL mixture. All samples were used in the bioassays against horn flies. 

**Figure 2:**
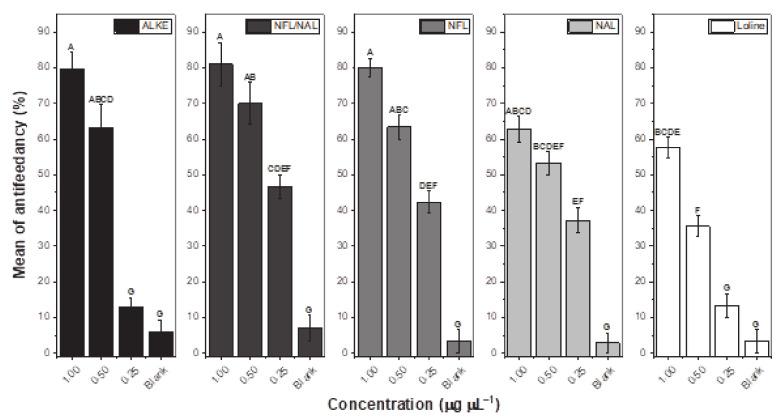
Antifeedancy levels of the alkaloid extract (ALKE) and lolines against horn flies at three concentrations (0.25, 0.50, and 1.00 µg µL^−1^) in laboratory bioassays compared to a blank. Values indicate the mean + SE. Letters on the columns indicate significant differences between the treatments and the blank based on the Tukey HSD test (*p* ≤ 0.05), *N* = 30.

In the antifeedant assay (Figure 2), all samples showed antifeedant activities and a concentration-dependent response was observed. Fly mortality was recorded during the bioassays, yet significant differences were not observed in relation to the control. Data analysis showed that the interaction among all the variables was significant (F = 5.41; df = 12, 40; *p* < 0.05). Particularly, some differences were observed among all alkaloidal samples against the blank (F = 22.2; df = 4, 40; *p* < 0.05) and among the three treatment doses (F = 298.75; df = 3, 40; *p* < 0.05). ALKE and loline significantly reduced the horn fly feeding by 63.1% and 35.5%, respectively, at 0.5 µg µL^−1^ concentration and 79.6% and 57.6%, respectively, at 1.0 µg µL^−1^ concentration. However, they were not antifeedant agents at 0.25 µg µL^−1^ when compared with the blank. The NFL/NAL mixture, NFL, and NAL significantly reduced the horn fly feeding at all doses evaluated. The mixture showed 80.9%, 70.0%, and 46.7% antifeedancy at 1.0, 0.5, and 0.25 µg µL^−1^, respectively; NFL elicited 80.1%, 63.3%, and 42.4% antifeedancy at 1.0, 0.5, and 0.25 µg µL^−1^, respectively; and NFL generated 62.8%, 53.3%, and 37.2% antifeedancy at 1.0, 0.5, and 0.25 µg µL^−1^, respectively (Figure 2). Comparing among treatments, less than 20.4% of the flies fed when they were exposed to ALKE, NAL/NFL mixture, or NFL at 1.0 µg µL^−1^, this being the major antifeedant activity. They did not differ significantly from the ALKE, NAL/NFL mixture, NFL, or NAL at 0.5 µg µL^−1^. On the contrary, 35.5% of flies did not feed when they were exposed to loline at 0.5 µg µL^−1^, being the less active treatment and equally significant to the NFL/NAL mixture, NFL, and NAL at 0.25 µg µL^−1^ and NAL at 0.5 µg µL^−1^. Among the pure lolines at 1.0 and 0.5 µg µL^−1^, NFL was the most active compound, being statistically equal to NAL but different to loline. However, NAL was not statistically different to loline. NFL and NAL at 0.25 µg µL^−1^ were statistically more active than loline. 

**Figure 3:**
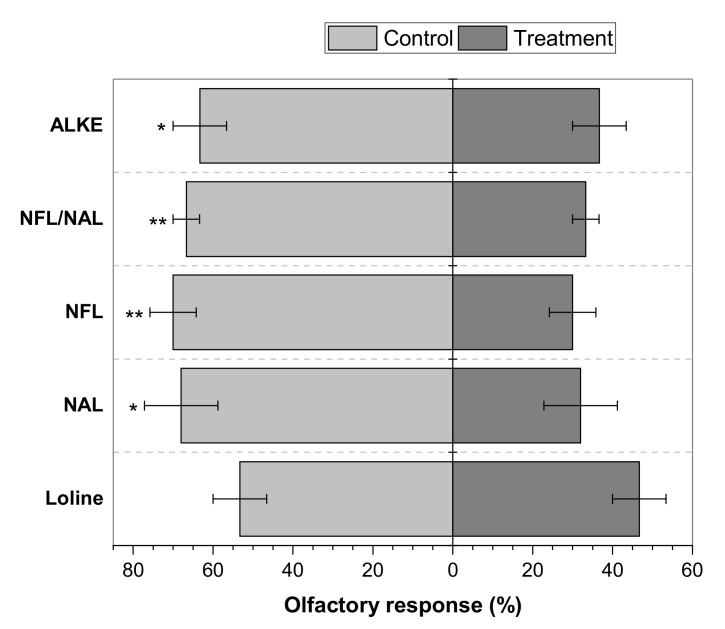
Olfactory response of horn flies to alkaloid extract and lolines. Values indicate the mean + SE. Asterisks on the bars indicate significant differences between the treatments and control based on the Tukey HSD test (* *p* ≤ 0.05, ** *p* ≤ 0.01), *N* = 60.

In the olfactory test, the Y-tube olfactometer allowed demonstration of the repellency provoked by four of the five alkaloidal samples tested (Figure 3). ALKE, the NAL/NFL mixture, NFL, and NAL exhibited spatial repellency in the olfactometer, where 63.3%, 66.7%, 70.0%, and 68.0% of the individuals chose the control, respectively. It was significantly different from the 33% and 30% of flies that chose the NFL/NAL mixture and NFL, respectively (*p* ≤ 0.01), and significantly different from the 36.7% and 32% of flies that chose ALKE and NAL, respectively (*p* ≤ 0.05). On the other hand, the 46.7% of the flies that chose loline did not differ significantly from the 53.3% of the flies that chose the control. Thus, loline was not a repellent agent against horn flies.

## 3. Discussion

The antifeedant effects and repellent activity of loline alkaloids against horn flies determined here appear not to be an exception to the anti-insect activities of lolines. Since the beginning of the 1990s, the insect dissuasion and insecticidal activity of loline alkaloids has been well documented [38,39,42,45,46,47,48,49,50,51]. Grass–endophyte symbiota, alkaloidal fractions containing lolines, and isolated lolines have been correlated with toxic, antifeedant, or repellent effects on a variety of insects in different biological stages. *Oncopeltus faciatus* (Hemiptera: Lygaeidae) [45], *Rhopalosiphum padi* (Hemiptera: Aphididae) [46,47], *Schizaphis graminum* (Hemiptera: Aphididae) [46,47,48], *Aulacorthum solani* (Hemiptera: Aphididae) [49], *Popillia japonica* (Coleoptera: Scarabaeidae) [50], *Spodoptera frugiperda* (Lepidoptera: Noctuidae) [48], *Ostrinia nubilalis* (Lepidoptera: Crambidae) [48], *Euplectrus comstockii*, and *Euplectrus plathypenae* (Hymenoptera: Eulophidae) [51] are some of species affected by loline alkaloids. In the studies performed by Siegel et al. [46] on *R. padi* and *S. graminum*, the loline concentrations in grass–endophyte plants were at least three times higher than the loline concentrations used in the present study. Also, lolines were always found to be associated with other anti-insect alkaloids. Similarly, in the studies on *S. frugiperda* and *O. nubilalis* [48] and *P. japonica* [50], the loline concentrations (≥ 500 ppm) producing antifeedant effects were higher than concentrations evaluated here. On the contrary, in the studies performed by Wilkinson et al. [47] against *R. padi* and *S. graminum*, the loline concentrations in plants were similar (ranging from 67 to 192 ppm) to the concentration used in the current study, except in one case where it was higher (ranging from 366 to 6060 ppm).

Despite the insect-deterring and insecticidal activity of loline alkaloids that has been described, only two studies have been published about the effects of loline alkaloids on *H. irritans* [38,39]. Dougherty et al. [38] determined that the mortality of *H. irritans* larvae in bovine dung that was supplemented with NFL was slightly higher than with NAL, whilst loline hydrochloride was not active. Although the presence of lolines in bovine dung from grazing animals has not been demonstrated [52,53], a higher activity of acyl loline derivatives than loline was established, as in the present case, where NFL and NAL were significantly more active than loline in antifeedant assays and olfactory responses. Also, Dougherty et al. [39] determined that ergotamine tartrate moderated the toxicity of NFL when *H. irritans* larvae were exposed to dung supplemented with a mixture of NFL and EAT, and that NFL was lethal for larvae, causing a linear dose-dependent reduction of the pupation. However, abnormalities of pupae or adults were not observed. It should be noted that just larvae and pupae *H. irritans* were the target in those previous studies [38,39]. Therefore, the present study is the first report about the effects of loline alkaloids against adult horn flies. Moreover, from studies performed by Dougherty et al. [38], Riedell et al. [48], and Schardl et al. [42], it can be clearly appreciated that 1-amine group substituents (Figure 1) are important in anti-insect activity, and the present study is no exception, as it has been demonstrated that the antifeedant activity of lolines was modulated by the substituent to the *N*-methyl group, whereas the lolines with a *N*-COH group, and to a lesser extent, with the *N*-COCH_3_ group, exhibited higher activity when compared to non-substituted loline. Despite this, the acyl loline isolation was not easy, time-consuming, and had poor yield, and considering that the performance of the 1:1 NFL/NAL mixture was statistically equal to or better than the acyl derivatives’ activity, it is advisable to use the mixture in future studies.

Deterrence and insecticidal effects of other natural substances against the adult stage of *H. irritans* have been reported [37,53,54,55,56,57,58,59,60,61,62,63,64,65,66,67,68]. Nevertheless, those studies have focused on establishing the toxic and repellent effects on horn flies. Only three studies about the antifeedant effect against horn flies have been published [54,55,56]. Showler and Harlien [54] reported that *p*-anisaldehyde completely deterred feeding from cotton pads soaked in bovine blood in response to 0.5 mL of *p*-anisaldehyde solutions at 0.6% up to 10% in acetone—doses almost 30 to 500 times higher than the maximum dose used in the present study—but horn flies were not repelled by *p*-anisaldehyde in static air tube olfactometers. On contrary, antifeedant effects were not detected using laboratory-grade limonene and a commercial limonene-based insecticide (Orange Guard) by the same bioassay [55], but those products attracted horn flies at concentrations <0.1%. Although in those studies, the reduction of the number of flies on the bloody pads in comparison to the blank was considered as an antifeedant effect, there was no evidence of blood consumption. In another study, Zhu et al. [56] reported that catnip oil, geraniol, and a 1:1:1 mixture of octanoic, nonanoic, and decanoic acids, collectively called “C8910 acids”, did reduce horn fly feeding in a laboratory bioassay and also exhibited spatial repellency in the olfactometer, where fewer than 15% of the flies fed when exposed to 0.2 or 2 mg of repellent. In the present study, ALKE, a 1:1 NFL/NAL mixture, NFL, and NAL reduced horn fly feeding, where fewer than 25% of the flies fed when exposed to 0.1 mg (100 µL of 1 µg µL^−1^ solution) of repellent (Figure 2), only half of the lower dose tested by Zhu et al. [56]. Mullens et al. [57] had previously tested the repellent effects of “C8910 acids” on horn flies based on reduction in horn fly fecal spots, where 1 mg cm^−2^ of “C8910 acids” repelled horn flies for at least 3 days in a laboratory assay. In the present study, the Y-tube olfactometer demonstrated repellency for all antifeedant agents, where over 63.3% of flies exposed to 10 µg of sample chose the control, showing less activity than the repellents used by Zhu et al. [56] at the same dose (>90% of flies chose the control).

In other Y-tube olfactometer studies, 2-decanone repelled horn flies at 1 µg and 0.01 µg, where over 70% of flies chose the control arm [58], but by using amounts 10 and 1000 times lower, respectively, than the dose used in the present study. Using a different apparatus, Klauck et al. [59] showed in an in vitro assay that 2 mL of 5% tea tree, *Melaleuca alternifolia* (Myrtales: Myrtaceae), and andiroba, *Carapa guianensis* (Sapindales: Meliaceae), essential oils (EOs) had a repellent effect on horn flies at 1 h and 2 h, respectively, after exposure. Additionally, both EOs (10 mL at 5%) demonstrated repellency at 24 h on infested cows, where 61.6% and 57.7% fewer flies, respectively, were on the treated cows than the control animals [60].

The strong anti-insect activities of loline alkaloids have been demonstrated. Although earlier studies with lolines have informed some in vitro vascular effects at extremely high concentrations [69,70], lolines could not be tied to toxicity to livestock and mammalian wildlife [42,43,44,71]. Current evidence using mice suggests that lolines have very low or non-toxicity on animals. In the 3-week experiment, the loline treatment caused no statistically significant effect on heart rate, blood pressure, motor coordination, haematological or serum biochemical parameters, or histopathological examination [44].

In summary, in the present study, all alkaloidal samples obtained directly and indirectly from *N. coenophialum*-infected tall fescue were antifeedant agents against horn flies, significantly reducing the horn fly feeding by between 35.5% and 80.9%. Additionally, ALKE, 1:1 NFL/NAL mixture, and both acyl derivatives were repellent agents of horn flies, producing over 63.3% repellency, and mortality of flies due to those antifeedant and repellent agents was negligible at the tested doses. Therefore, the minor or non-adverse effects for cattle and beneficial activities of those lolines make them suitable candidates for horn fly management, although further field studies related to the antifeedancy, toxicity, and repellence promoted by loline alkaloids are necessary.

## 4. Materials and Methods

### 4.1. H. irritans Collection

Laboratory bioassays were conducted using wild horn flies collected from beef cattle according to Oyarzún et al. [58], with modifications. Fifteen steers fed on endophyte-free tall fescue pastures were placed in a corridor at Centro Regional de Investigación INIA Carillanca, Vilcún, Chile (38°41′ SL, 72°25′ WL, 200 masl). Then, horn flies feeding on steers were hand-collected and trapped in a 1 L glass flask per bovine (50–60 insects per flask), which were subsequently sealed with an entomological net and were transported to the laboratory. Once in the laboratory, the horn flies were pooled together and maintained at 25 ± 2 °C under a light:dark photoperiod of 12:12 h with variable humidity (30–50%).

### 4.2. Plant Material

*Festuca arundinacea* cultivar K-31 plants infected with *Neotyphodium coenophiaum* were collected in December 2019 from an experimental fescue pasture located at Centro Regional de Investigación INIA Carillanca, Vilcún, Chile (38°41′ SL, 72°25′ WL, 200 masl). The endophytic fungi presence in the cultivar was checked by the method described by Belanger [72] and Saha et al. [73] Briefly, tissues of the bark, sheath, leaves, and stem were placed on a glass slide. Then, 1–2 drops of rose bengal staining solution (0.5% rose bengal dissolved in 5% aqueous solution of ethanol (*w/v*) [73]) were applied to the sample for 30–60 s, and it was covered with a coverslip. The stained tissues were examined microscopically (400×). A positive result was considered when association between plants and hyphae in the region of the aleurone layer was observed.

### 4.3. Alkaloid Extract Obtention

Oven-dried and milled plant material (300 g) was defatted with petroleum ether (35–60 °C) and extracted with methanol using a Soxhlet apparatus over a period of 8 h. The suspension that was obtained was filtered through filter paper, and the resulting methanolic extract was evaporated under reduced pressure on a rotatory evaporator. The syrupy residue was agitated with 250 mL 0.75% HCl for 1 h at 35 °C and filtered through a frit funnel. The clear filtrate was washed with CH_2_Cl_2_ (5 × 20 mL). The aqueous phase was adjusted to pH 10 with NH_4_OH and extracted with CH_2_Cl_2_ until the extracts gave a negative Dragendorff reaction. Finally, the organic layer was dried with anhydrous Na_2_SO_4_ and the evaporation of the solvent yielded an orange-brown oil corresponding to the alkaloid extract (ALKE) (0.2378 g).

### 4.4. N-Formyl Loline and N-Acetyl Loline Isolation

In a 10 mL flask, the alkaloid extract (0.1824 g) was diluted in CH_2_Cl_2_ (4 mL) and the solution was seeded onto a 20 × 20 cm preparative glass chromatoplate coated with 1 mm silica gel (60_F254_, Merck, Darmstadt, Germany). The plates were developed three times using CH_2_Cl_2_-MeOH-NH_4_OH (49.5:49.5:1.0) and examined under UV light at 254 nm. Dragendorff reagent was used to assess for the presence of alkaloids. The alkaloid-containing bands of Rf = 0.70 were removed from the chromatoplate and extracted using CH_2_Cl_2_–MeOH (7:3). Subsequently, the suspension was vacuum-filtered, dried with anhydrous sodium sulfate, filtered, and evaporated to dryness using a rotatory evaporator to yield a yellow oil corresponding to a mixture of *N*-formyl loline and *N*-acetyl loline (NFL/NAL 1:1; 0.092 g). The NFL/NAL mixture (0.060 g) was separated by column chromatography (CC) using silica gel 60 (70–230 mesh ASTM, Merck KGaA, Darmstadt, Germany). The CC was eluted using a CH_2_Cl_2_–MeOH step gradient. The alkaloids in each fraction were obtained after having been dried with anhydrous sodium sulfate, filtered, and evaporated to dryness by rotatory evaporator to yield two pale-yellow oils corresponding to *N*-formyl loline (0.0048 g) and *N*-acetyl loline (0.0069 g). The products were analyzed by GC/MS (NFL RT: 12.81 min; MS data (*m/z*): 182.0 [M]^+^, 167.5, 153.9, 137.9, 123.1, 109.9, 94.9, 81.8 base pick; NAL RT: 14.16 min; MS data (*m/z*): 195.9 [M]^+^, 166.9, 152.9, 137.9, 122.9, 109.9, 94.9, 81.8 base pick) and they were compared with a commercial standard of *N*-formyl loline and *N*-acetyl loline (AgResearch, Hamilton, New Zealand).

### 4.5. Loline Preparation

In a 5 mL round-bottom flask, an NFL/NAL mixture (1:1) (0.031 g; NAL: 79.0 µmol, NFL: 85.1 µmol) was treated with 5% HCl (1 mL) and sonicated at 60 °C for 4 h. After, the reaction mixture was alkalinized up to pH 12 with 37% NH_4_OH. The mixture was suspended in CH_2_Cl_2_ (5 mL) and successively extracted with CH_2_Cl_2_ (3 × 5 mL). The organic layer was dried with anhydrous sodium sulfate, filtered, and evaporated to dryness using a rotatory evaporator to yield a pale-yellow oil corresponding to loline (8.6 mg; 55.8 µmol; 35.3%). The product was analyzed by GC/MS (RT: 5.10 min; MS data (*m/z*): 153.9 [M]^+^, 136.9, 122.9, 109.6, 94.9, 81.8 base pick) and it was compared with a commercial standard of loline (Chemfaces, Wuhan, China).

### 4.6. Analyses of Loline Alkaloids by GC/MS

The analyses of the alkaloid extract (ALKE) and lolines were performed by GC/MS, using the following instrumentation: a Thermo Electron Model Focus GC (Waltham, MA, USA) coupled to a DSQ Thermo Electron quadrupole mass spectrometric detector, with an integrated data system (Xcalibur 2.0, Thermo Fisher Scientific Inc., Waltham, MA, USA) and a 30 m length BPX5 capillary column (0.25 µm film thickness × 0.25 mm i.d., SGE Forte, Trajan Scientific and Medical, Ringwood, Victoria, Australia). The operating conditions were as follows: on-column injection; injector, transfer line, and detector temperature, 250 °C; oven temperature program: 110 °C increased to 188 °C at 4 °C min^−1^, and then maintained for 5 min. Subsequently, it was increased to 250 °C at 10 °C min^−1^, and finally maintained at 250 °C for 5 min. He at 1.00 mL min^−1^ was used as carrier gas. The mass spectra were obtained at an ionization voltage of 70 eV. The recording conditions employed a scan time of 1.5 s and a mass range of 30 to 400 amu. The compounds were identified comparing their retention times and mass spectra with those of commercial standards (loline: Chemfaces, Wuhan, China; NFL and NAL: AgResearch, Hamilton, New Zealand).

### 4.7. Laboratory Feeding Bioassay

#### 4.7.1. Blood Collection

The blood samples (4.5 mL per bovine) obtained from 15 steers fed on endophyte-free tall fescue pastures were collected by puncture of the jugular vein, with the use of 13 × 75 mm, 4.5 mL BD Vacutainer^®^ glass tubes (Becton Dickinson, Franklin Lakes, NJ, USA) containing buffered sodium citrate solution (3.2%, 0.109 M). The blood samples were transferred to the laboratory in coolers at a temperature close to 4–7 °C. Once in the laboratory, the blood samples were pooled together in a sterile glass flask and kept at 4 °C.

#### 4.7.2. Feeding Bioassay

The feeding behavior of horn flies was tested based on a no-choice test according to Zhu et al. [56] with modifications. Test flies were starved for 24 h prior to testing. Doses (100 μL) of ALKE and lolines dissolved in acetone (liquid chromatography grade; LiChrosolv^®^, Merck KGaA, Darmstadt, Germany) at 0.25, 0.50, and 1.00 µg µL^−1^ concentrations were equally applied to the tulle piece (4 × 5 cm). After the solvent evaporated (2–3 min), the sample-impregnated layer was placed on top of a blood-soaked cotton pad and it was put into a glass flask (4 × 5 × 6.45 cm). A control sample was treated with 100 μL of acetone only. Test flies were transferred into each testing flask (10 flies per flask). After 4 h, the mortality was recorded, and the flies were frozen at −20 °C for 1 h. They were then checked for feeding status by squashing their abdomens and examining under microscope (20×) for the presence of blood. The experiments were performed in triplicate. The antifeeding performance was evaluated according to Equation (1):

(1) $$Mean\;of\;antifeedancy(\%)=\frac{\left(Unfed\;flies\times 100\right)}{Total\;flies}$$

### 4.8. Olfactometer Bioassays

A dual-port Y-tube glass olfactometer (stem, 110 mm; ports, 90 mm at a 130° angle from the stem; internal diameter, 10 mm) was used to assess the olfactory response of *H. irritans* [58] to loline alkaloids. Each port of the Y-tube was connected to a Pasteur pipette, each containing either the sample or the control. A quantity of 10 μL of sample, 1 μg μL^−1^ in acetone, was applied to a piece of filter paper (0.5 cm × 5 cm). For the control, 10 μL of acetone was applied. The filter paper was air-dried and placed in the middle of a Pasteur pipette connected to a port of the olfactometer. The base of the stem was connected to a vacuum pump generating a purified air flow (800 mL min^−1^) to carry the volatile stimuli from the ports to the stem. The air was purified through a column of activated carbon. Unsexed horn flies were starved for 24 h prior to testing. To test the fly response to samples, flies were released into the olfactometer individually through a hole at 2 cm of the base of the stem, and immediately afterward it was sealed with a Teflon cap and given 3 min for choosing between the sample or control. Their presence in the sample-treated or control port (>half of the port) was recorded. If the fly did not make any choice, it was discarded. Each sample was tested with 10 different flies, sequentially introduced into the olfactometer. After two flies were tested, the Y-tube was cleaned with ethanol followed by acetone, and air-dried. Also, the order of ports was randomized. Each test was replicated 6 times using 10 different flies each time. Responses were recorded as the percentage of flies inside the treatment or control ports.

### 4.9. Statistical Analyses

The statistical software Statistix 10 (Analytical Software, Tallahassee, FL, USA) was used to analyze the data. The Shapiro–Wilk test was used to test whether data conformed to a normal distribution. The differences in the antifeedant activity and olfactory response among treatments on *H. irritans* were analyzed using a two-way ANOVA test (*p* ≤ 0.05) with a post-hoc Tukey HSD test. Olfactory response among treatments on *H. irritans* were analyzed using a one-way ANOVA test (*p* ≤ 0.05, *p* ≤ 0.01) with a post-hoc Tukey HSD test. The results were expressed as means with their corresponding standard errors. 

## Data Availability

Data sharing is not applicable to this article.

## Sample Availability

Samples of the compounds are not available from the authors.

## References

[1] Cupp E.W., Cupp M.S., Ribeiro J.M., Kunz S.E. (1998). Blood-feeding strategy of *Haematobia irritans* (Diptera: Muscidae). J. Med. Entomol..

[2] Kuramochi K. (2000). Survival, ovarian development and bloodmeal size for the horn fly *Haematobia irritans irritans* reared in vitro. Med. Vet. Entomol..

[3] Mancebo A., Monzón C.M., Bulman G.M. (2001). *Haematobia irritans*: Una actualización a diez años de su introducción en Argentina (Parte I). Vet. Argent..

[4] Mancebo A., Monzón C.M., Bulman G.M. (2001). *Haematobia irritans*: Una actualización a diez años de su introducción en Argentina (Parte II). Vet. Argent..

[5] Foil L.D., Hogsette J.A. (1994). Biology and control of tabanids, stable flies and horn flies. Rev. Sci. Tech..

[6] Quiroz R.H. (2005). Parasitología y Enfermedades Parasitarias de los Animales Domésticos.

[7] James M.T., Harwood R.F. (1969). Herms’ Medical Entomology.

[8] Depner K.R. (1961). The effect of temperature on development and diapause of the horn fly, *Siphona irritans* (L.) (Diptera: Muscidae). Can. Entomol..

[9] Carballo M., Martínez M. (1991). Hallazgo de *Haematobia irritans* en Uruguay. Veterinaria.

[10] Luzuriaga R., Eddi C., Caracostantogolo J., Botto E., Pereira J. (1991). Diagnosis of infestation of cattle by *Haematobia irritans* (L.) in Misiones, Argentina. Rev. Med. Vet..

[11] Cisternas E.A. (1999). Mosca de los cuernos *Haematobia irritans*. Inf. INIA Remehue.

[12] Fundación para la Innovación Agraria (FIA), BTA Consultores (2009). Resultados y Lecciones en Control. Biológico de la Mosca de los Cuernos en bovinos con Extracto de Neem: Proyecto de Innovación en regiones Metropolitana, de Valparaíso, del Maule y del Biobío.

[13] Schreiber E.T., Campbell J.B., Kunz S.E., Clanton D.C., Hudson D.B. (1987). Effects of horn fly (Diptera: Muscidae) control on cows and gastrointestinal worm (Nematode: Trichostrongylidae) treatment for calves on cow and calf weight gains. J. Econ. Entomol..

[14] DeRouen S.M., Foil L.D., MacKay A.J., Fraenke D.E., Sanson D.W., Wyatt W.E. (2003). Effect of horn fly (*Haematobia irritans*) control on growth and reproduction of beef heifers. J. Econ. Entomol..

[15] Campbell J.B. (1976). Effect of horn fly control on cows as expressed by increased weaning weights of calves. J. Econ. Entomol..

[16] Harvey T.L., Brethour J.L. (1979). Effect of horn flies on weight gains of beef cattle. J. Econ. Entomol..

[17] Steelman C.D., Brown A.H., Gbur E.E., Tolley G. (1991). Interactive response of the horn fly (Diptera: Muscidae) and selected breeds of beef cattle. J. Econ. Entomol..

[18] Foil L.D., Allison M., DeRouen S.M., Kimball M., Morrison D.M., Sanson D.W., Wyatt W.E. (2000). Controlling horn flies. La Agric..

[19] Williams J.D., Sutherst R.W., Maywald G.F., Petherbridge C.T. (1985). The southward spread of buffalo fly (*Haematobia irritans exigua*) in eastern Australia and its survival through a severe winter. Aust. Vet. J..

[20] Metcalf R.L., Metcalf R.A. (1993). Destructive and Useful Insects.

[21] Hawkins J.A. (2001). The Horn Fly (Haematobia irritans).

[22] DeRouen S.M., Miller J.E., Foil L.D. (2010). Control of horn flies (*Haematobia irritans*) and gastrointestinal nematodes and its relation with growth performance in stocker cattle. Prof. Anim. Sci..

[23] Grisi L., Cerqueira R., de Souza J., Medeiros A., Andreotti R., Duarte P., Pérez A., Barros J., Silva H. (2014). Reassessment of the potential economic impact of cattle parasites in Brazil. Braz. J. Vet. Parasitol..

[24] Velasco R., González J., Morales G., Ortega E. (2001). Daño económico y costos de control en bovinos mosca de los cuernos. Inf. Agropecu. Bioleche INIA Quilamapu.

[25] Lanuza F., Paredes E., Sievers G., Cortázar J.M., Catrileo A. (2005). Manejo sanitario y principales enfermedades de los bovinos de carne. Producción y Manejo de Carne Bovina en Chile.

[26] Guglielmone A.A., Castelli M.E., Idiart J., Fisher W.F., Volpogni M.M., Quaino O., Anziani O.S., Flores S.G., Warnke O. (1999). Skin lesions and cattle hide damage from *Haematobia irritans* in cattle. Med. Vet. Entomol..

[27] Barros A.T.M., Ottea J., Sanson D., Foil L.D. (2001). Horn fly (Diptera: Muscidae) resistance to organophosphate insecticides. Vet. Parasitol..

[28] Oyarzún M.P., Quiroz A., Birkett M.A. (2008). Insecticide resistance in the horn fly: Alternative control strategies. Med. Vet. Entomol..

[29] Oyarzún M.P., Li Y., Figueroa C.C. (2011). High levels of insecticide resistance in introduced horn fly (Diptera: Muscidae) populations and implications for management. J. Econ. Entomol..

[30] Parra L., Rojas C., Catrileo A., Galdames R., Mutis A., Birkett M.A., Quiroz A. (2013). Differences in the fly-load of *Haematobia irritans* (Diptera: Muscidae) on cattle is modified by endophyte infection of pastures. Electron. J. Biotechnol..

[31] Parra L., Mutis A., Chacón M., Lizama M., Rojas C., Catrileo A., Rubilar O., Tortella G., Birkett M.A., Quiroz A. (2016). Horn fly larval survival in cattle dung is reduced by endophyte infection of tall fescue pasture. Pest. Manag. Sci..

[32] Hoveland C.S. (1993). Economic importance of Acremonium endophytes. Agric. Ecosyst. Environ..

[33] Schardl C.L., Leuchtmann A., Spiering M.J. (2004). Symbioses of grasses with seedborne fungal endophytes. Annu Rev. Plant. Biol..

[34] Potter D.A., Tyler J.T., Redmond C.T., Schardl C.L., Panaccione D.G. (2008). Contribution of ergot alkaloids to suppression of a grassfeeding caterpillar assessed with gene knockout endophytes in perennial ryegrass. Entomol. Exp. Appl..

[35] Panaccione D., Beaulieu W., Cook D. (2014). Bioactive alkaloids in vertically transmitted fungal endophytes. Funct. Ecol..

[36] Schardl C.L., Young C.A., Faulkner J.R., Florea S., Pan J. (2011). Chemotypic diversity of epichloae, fungal symbionts of grasses. Fungal Ecol..

[37] Showler A.T. (2017). Botanically based repellent and insecticidal effects against horn flies and stable flies (Diptera: Muscidae). J. Integr. Pest. Manag..

[38] Dougherty C.T., Knapp F.W., Bush L.P., Maul J.E., van Willigen J. (1998). Mortality of horn fly (Diptera: Muscidae) larvae in bovine dung supplemented with loline alkaloids from tall fescue. J. Med. Entomol..

[39] Dougherty C.T., Knapp F.W., Bush L.P. (1999). Mortality of horn fly larvae (Diptera: Muscidae) in bovine dung supplemented with ergotamine and N-formyl loline. J. Med. Entomol..

[40] Faeth S.H. (2002). Are endophytic fungi defensive plant mutualists?. Oikos.

[41] Nelli M.R., Scheerer J.R. (2016). Synthesis of Peramine, an Anti-insect Defensive Alkaloid Produced by Endophytic Fungi of Cool Season Grasses. J. Nat. Prod..

[42] Schardl C.L., Grossman R.B., Nagabhyru P., Faulkner J.R., Mallik U.P. (2007). Loline alkaloids: Currencies of mutualism. Phytochemistry.

[43] Popay A., White J., Torres M. (2009). Insect herbivory and defensive mutualisms between plants and fungi. Defensive Mutualism in Microbial Symbiosis.

[44] Finch S., Munday J., Munday R., Kerby J. (2016). Short-term toxicity studies of loline alkaloids in mice. Food Chem. Toxicol..

[45] Yates S.G., Fenster J.C., Bartelt R.J. (1989). Assay of tall fescue seed extracts, fractions, and alkaloids using the large milkweed bug. J. Agric. Food Chem..

[46] Siegel M.R., Latch G.C.M., Bush L.P., Fannin F.F., Rowan D.D., Tapper B.A., Bacon C.W., Johnson M.C. (1990). Fungal endophyte-infected grasses: Alkaloid accumulation and aphid response. J. Chem. Ecol..

[47] Wilkinson H.H., Siegel M.R., Blankenship J.D., Mallory A.C., Bush L.P., Schardl C.L. (2000). Contribution of fungal loline alkaloids to protection from aphids in a grass-endophyte mutualism. Mol. Plant. Microbe. Interact..

[48] Riedell W.E., Kieckhefer R.E., Petroski R.J., Powell R.G. (1991). Naturally occurring and synthetic loline alkaloid derivatives: Insect feeding behavior modification and toxicity. J. Entomol. Sci..

[49] Lehtonen P., Helander M., Wink M., Sporer F., Saikkonen K. (2005). Transfer of endophyte-origin defensive alkaloids from a grass to a hemiparasitic plant. Ecol. Lett..

[50] Patterson C.G., Potter D.A., Fannin F.F. (1991). Feeding deterrency of alkaloids from endophyte-infected grasses to Japanese beetle grubs. Entomol. Exp. Appl..

[51] Bultman T.L., Borowicz K.L., Schneble R.M., Coudron T.A., Bush L.P. (1997). Effect of a fungal endophyte on the growth and survival of two *Euplectrus* parasitoids. Oikos.

[52] Westendorf M.L., Mitchell G.E., Tucker R.E., Bush L.P., Petroski R.J., Powell R.G. (1993). In Vitro and in Vivo Ruminal and Physiological Responses to Endophyte-Infected Tall Fescue. J. Dairy Sci..

[53] Espinoza J., Ungerfeld E., Escobar P., Piñeira J., Mejías J., Quiroz A., Reyes M., Luengo A. (2017). Loline alkaloids presence in blood serum, urine and feces of steers fed with tall fescue (*Festuca arundinacea*) infected by endophytic fungus *Neotyphodium coenophialum*. 6th International Workshop in Advances in Science and Technology of Bioresources, Pucón, Chile, 29–30 November–01 December 2017.

[54] Showler A.T., Harlien J.L. (2018). Effects of the botanical compound *p*-anisaldehyde on horn fly (Diptera: Muscidae) repellency, mortality, and reproduction. J. Med. Entomol..

[55] Showler A.T., Harlien J.L., Perez de Léon A.A. (2019). Effects of Laboratory Grade Limonene and a Commercial Limonene-Based Insecticide on *Haematobia irritans* irritans (Muscidae: Diptera): Deterrence, Mortality, and Reproduction. J. Med. Entomol..

[56] Zhu J.J., Brewer G.J., Boxler D.J., Friesen K., Taylor D.B. (2015). Comparisons of antifeedancy and spatial repellency of three natural product repellents against horn flies, *Haematobia irritans* (Diptera:Muscidae). Pest. Manag. Sci..

[57] Mullens B.A., Reifenrath W.G., Butler S.M. (2009). Laboratory trials of fatty acids as repellents or antifeedants against house flies, horn flies and stable flies (Diptera: Muscidae). Pest. Manag. Sci..

[58] Oyarzún M.P., Palma R., Alberti E., Hormazabal H., Pardo F., Birkett M.A., Quiroz A. (2009). Olfactory Response of *Haematobia irritans* (Diptera: Muscidae) ton Cattle-Derived Volatile Compounds. Med. Entomol..

[59] Klauck V., Pazinato R., Radavelli W.M., Volpato A., Stefani L.M., Santos R.C., Vaucher R.A., Boligon A.A., Athayde M.S., da Silva A.S. (2015). In vitro repellent effect of tea tree (*Melaleuca alternifolia*) and andiroba (*Carapa guianensis*) oils on *Haematobia irritans* and *Chrysomya megacephala* flies. Trop. Biomed..

[60] Klauck V., Pazinato R., Stefani L.M., Santos R.C., Vaucher R.A., Baldissera M.D., Raffin R., Boligon A., Athayde M., Baretta D. (2014). Insecticidal and repellent effects of tea tree and andiroba oils on flies associated with livestock. Med. Vet. Entomol..

[61] Marlatt C.L. (1928). Report of the Entomologist.

[62] Stanbury R.E., Goodhue L.D. (1960). New fly repellents in dairy sprays. Agric. Chem..

[63] Heimerdinger A., Olivo C.J., Sobczak M.F., Gabbi A.M., Scaravelli L.F.B., Pereira L.E.T., Piuco M.A. (2004). Utilization of Phytotherapetic Products on the Control of Haematobia Irritans in Dairy Cows Contaminated Naturally.

[64] Milián C.A.F. (2009). Evaluación del Efecto Repelente del té de Limón (*Cymbopogon citratus*) Utilizado a Diferentes Diluciones Contra la Mosca los Cuernos (*Haematobia irritans*) en Ganado Bovino de Doble Propósito Durante el Mes de Entere de 2008 en Salamá, Baja Verapaz. Licentiate Thesis.

[65] Ramirez A.M., Carillo A.C., Molano C.R. (2009). Preliminary screening of ethanolic extracts of five medicinal plants against *Haematobia irritans* L. (Diptera: Muscidae). Rev. UDCA Actual. Divulg. Cient..

[66] Juan L.W., Lucia A., Zerba E.N., Harrand L., Marco M., Masuh H.M. (2011). Chemical composition and fumigant toxicity of the essential oils from 16 species of Eucalyptus against *Haematobia irritans* (Diptera: Muscidae) adults. J. Econ. Entomol..

[67] Lachance S., Grange F. (2014). Repellency effectiveness of seven plant essential oils, sunflower oil and natural insecticides against horn flies on pastured dairy cows and heifers. Med. Vet. Entomol..

[68] Mullens B.A., Watson D.W., Gerry A.C., Sandelin B.A., Soto D., Rawls D., Denning S., Guisewite L., Cammack J. (2017). Field trials of fatty acids and geraniol applied to cattle for suppression of horn flies, *Haematobia irritans* (Diptera: Muscidae), with observations on fly defensive behaviors. Vet. Parasitol..

[69] Oliver J.W., Strickland J.R., Waller J.C., Fribourg H.A., Linnabary R.D., Abney L.K. (1998). Endophytic fungal toxin effect on adrenergic receptors in lateral saphenous veins (cranial branch) of cattle grazing tall fescue. J. Anim. Sci..

[70] Solomons R.N., Oliver J.W., Linnabary R.D. (1989). Reactivity of dorsal pedal vein of cattle to selected alkaloids associated with *Acremonium coenophialum*-infected fescue grass. Am. J. Vet. Res..

[71] Rudolph W., Remane D., Wissenbach D.K., Peters F.T. (2019). Liquid chromatography-mass spectrometry-based determination of ergocristine, ergocryptine, ergotamine, ergovaline, hypoglycin A, lolitrem B, methylene cyclopropyl acetic acid carnitine, *N*-acetylloline, *N*-formylloline, paxilline, and peramine in equine hair. J. Chromatogr. B.

[72] Belanger F.C. (1996). A rapid seedling screening method for determination of fungal endophyte viability. Crop. Sci..

[73] Saha D.C., Jackson M.A., Johnson-Cicalese J.M. (1988). A rapid staining method for detection of endophytic fungi in turf and forage grasses. Phytopathology.

